# Exploring Barriers and Facilitators to Patients and Members of the Public Contributing to Rapid Health Technology Assessments for NICE: A Qualitative Study

**DOI:** 10.1111/hex.70109

**Published:** 2024-11-18

**Authors:** Eugenie Evelynne Johnson, Debbie Smith, Becky Harmston, Emily Hunter, Emma Belilios, Fiona Pearson

**Affiliations:** ^1^ Population Health Sciences Institute Newcastle University Newcastle upon Tyne UK; ^2^ NIHR Innovation Observatory Newcastle University Newcastle upon Tyne UK; ^3^ Public Involvement Member UK; ^4^ Newcastle upon Tyne Hospitals NHS Foundation Trust Newcastle upon Tyne UK

**Keywords:** Health Technology Assessment, patient and public involvement, patient engagement, patient involvement

## Abstract

**Background:**

Evidence and External Assessment Groups (EAGs) assist in the National Institute of Health and Care Excellence's Technology Appraisal programme by either critiquing evidence provided by companies on different health technologies, or by carrying out an independent search and evaluation of the published evidence. Historically, there has been little patient and public involvement within the work of EAGs.

**Objective:**

To identify key barriers and facilitators to patient and public involvement in EAG Reports feeding into the National Institute for Health and Care Excellence's Health Technology Appraisal process.

**Methods:**

A primary qualitative study consisting of one‐to‐one interviews with EAG researchers and focus groups with members of the public. From anonymised transcripts, data were deductively coded using a framework analysis against the Theoretical Domains Framework and translated to the COM‐B model. Coding was triangulated through inductive thematic analysis, guided by the principles of Braun and Clarke.

**Results:**

Ten researchers were interviewed and four focus groups with a total of 26 members of the public were undertaken. Both EAG researchers and the public felt they did not have enough knowledge, time and money to be able to embed patient and public involvement; researchers suggested that patient and public involvement might not be relevant to the scope of their Reports. Members of the public highlighted a lack of awareness of the Technology Appraisal process and that jargon may stop them being involved. Both researchers and members of the public said having specific guidance on how to embed patient and public involvement in EAG Reports would be helpful, including guidance on how to write plain language summaries.

**Conclusion:**

The perspectives of both EAG researchers and members of the public suggest work needs to be conducted to produce frameworks for patient and public involvement and plain language summaries within EAG Reports specifically. Additionally, that further awareness‐raising of Technology Appraisals and the role of EAGs would help members of the public to contribute effectively to EAG Reports.

**Patient or Public Contribution:**

Two members of the public were part of the research team and governed all stages of the research in accordance with the UK Standards for Public Involvement.

## 1 Introduction

In the United Kingdom, the National Institute for Health and Care Excellence (NICE) produce Technology Appraisals and Assessments (TAs) that present recommendations on the use of health technologies (medicines, devices and diagnostic tools) within the National Health Service (NHS) [1]. Evidence and External Assessment Groups (EAGs) contribute to the TA process either by producing a rapid critical appraisal of clinical and cost‐effectiveness evidence on a health technology produced by a company, or by undertaking an independent literature search and critique of the clinical and economic literature base (including evidence synthesis and meta‐analysis, where appropriate). Some EAG Reports, such as those produced for Single Technology Assessments, are carried out within 8 weeks.

There is an expectation from the National Institute for Health and Care Research (NIHR) that patient and public involvement (PPI) is embedded into healthcare research [2]. Patient and public members contribute to NICE's TA process by providing comments on draft guidance consultations, being Committee members and observing committee meetings [3]. Voluntary and charitable sector organisations also provide evidence submissions. However, historically, patient and public members do not contribute directly to EAG Reports contributing to the TA process.

What can be considered meaningful PPI within EAG Reports is challenging, particularly as there is currently no framework or guidance outlining what is considered “good practice” in this context. For example, NIHR INCLUDE is focused on participation in clinical trials [4], whereas EAG Reports are not focused on conducting primary research. Furthermore, the Authors and Consumers Together Impacting on eVidencE (ACTIVE) framework for PPI in evidence synthesis does not consider how PPI can be conducted at the rapid pace required to produce EAG Reports [5].

However, there is currently little known about why researchers working on EAG Reports do, or do not, engage and consult patients and the public. Similarly, we know very little about why patients and members of the public may, or may not, wish to be involved in contributing their perspectives to EAG Reports. Before any guidance regarding how PPI can be embedded within EAG Reports is produced, we need to know more about the reasons both why EAG researchers may or may not embed PPI in their reports and what may prevent or aid patients and the public in being involved. Therefore, this qualitative study forms a preliminary exploration of these factors from patient, public and researcher perspectives.

## 2 Materials and Methods

### 2.1 Ethical Approval

Ethical approval for this study was obtained from Newcastle University on August 23, 2023 (reference 2600/34662).

### 2.2 Public Involvement

The role of the public researchers (D.S. and B.H.) was guided by the principles of the UK Standards for Public Involvement [6, 7]. A summary of the Standards and how we achieved these within this work is described in Table 1.

**Table 1:** UK Standards for Public Involvement in relation to work with the public researchers.

UK Standard	Summary of standard	Example activities
Inclusive opportunities	Providing public involvement opportunities that are accessible and reach people according to research need	− Open advertising of the opportunity to be part of the NIHR Innovation Observatory Public Advisory Group (*n* = 19) − Offering recognition payments for time spent working on the project
Working together	Working together in a way that values contributions, builds and sustains mutual respect and productive relationships	− Ensuring the purpose of the public governance role was clear and outlining expectations of both the public members and researchers before project start − Involving the public members in different aspects of the project, including decision making in team meetings, shaping the workshops, commenting on findings, contributing to final report and dissemination − Recognising the role the public governance members had in shaping the project
Support and learning	Offering and promoting support and learning opportunities to build confidence and skills for involvement in research	− Inducting the public governance members into the project and asking about potential areas for development within this − Offering ad hoc support to public governance members, where desired − Evaluating the experience of the public governance members to identify what went well and what did not, to take to future projects
Communications	Using plain language for well‐timed, relevant communications	− Flexibility in communication methods, where desired (e.g., email or online meetings) − Inclusion in research team correspondence, such as notes and actions from project meetings
Impact	Identifying and sharing the difference public involvement makes to research	− Clear signposting of the contributions of the public governance team members within the project final report − Reflecting on and evaluating the involvement of the public governance members − Use of the PIRIT toolkit to demonstrate the impact of PPI throughout the project [8]
Governance	Involving the public in the management, regulation, leadership and decision making within research	− Inclusion of public governance team members in team meetings, correspondence and the design and delivery (where wanted) of the project − Defined role of the public contributors; expectations of the role discussed and agreed within an informal induction − Resources in place to provide timely recognition payments for time spent working on the project; flexibility in how these were delivered

*Note:* Adapted from Crowe et al. [6, 7].

Abbreviations: NIHR, National Institute for Health and Care Research; PIRIT, Patient Involvement in Research Impact; UK, United Kingdom.

### 2.3 Theoretical Framework

As the purpose of the study was to identify barriers and facilitators, we implemented an exploratory design, whereby participants' views are presented without interpretation. Since an exploratory design seeks to be descriptive in nature, this study did not adopt a specific methodological viewpoint [9].

### 2.4 Participants and Recruitment

We designed two information sheets and consent forms specifically targeting either EAG researchers or patients and members of the public using the secure web platform Qualtrics [10]. The information sheets clearly explained the purpose of the project, what the interviews or focus groups would consist of, information regarding data handling and how to withdraw, and an email address to contact. These also contained a consent form to participate. The information sheets can be found in Supplementary Material S1 and S2.

To recruit members of the public for the focus groups, we distributed the information sheet to organisations with an interest in public and patient involvement and advocacy, posted on the VOICE global platform and shared through the NIHR Innovation Observatory social media and to the NIHR Innovation Observatory Public Advisory Group, as well as via the NICE Patient Involvement Programme team. We asked organisations to cascade the information sheet via newsletters or through snowballing to anyone with a potential interest. Our public governance members were asked to snowball to anyone they felt may be interested in the focus groups. For the one‐to‐one interviews with researchers, we sent the information sheet and consent form directly to independent research groups listed on a publicly available list of EAGs published by the NIHR [11].

For the focus groups, we took a purposive sampling approach using the demographic information provided within the consent forms (geographic location within the United Kingdom, age range and ethnic group). One researcher (E.E.J.) selected up to eight participants for each focus group with the intention of enabling a diverse range of perspectives to be captured at each focus group. The only eligibility criteria for the interviews with researchers was that they needed to have been involved in at least one EAG Report in the past. To avoid potential conflict of interest, we did not contact the EAGs based at Newcastle University and the Newcastle upon Tyne Hospitals NHS Foundation Trust regarding participation.

### 2.5 Data Collection

We conducted all interviews via Microsoft Teams and all focus groups via Zoom. The interviews could last for up to an hour and were designed to be semistructured to allow for discussion and elaboration on both barriers and facilitators to PPI in EAG Reports. The topic guide for interviews can be found in Supplementary Material S3. We created a similar semistructured topic guide for the focus groups, which were scheduled for an hour each. Additionally, one researcher (E.E.J.) created vignettes using the online platform Canva Pro to explain two hypothetical narratives surrounding imagined researchers in EAGs, patients and carers to elicit responses where there may have been a lack of knowledge of the report production process [12]. The public researchers (D.S. and B.H.) commented on and refined these. The final semistructured topic guide for the focus groups is presented in Supplementary Material S4 and screenshots of the vignettes used are shown in Supplementary Material S5. One of two researchers (E.E.J. or E.H.) carried out the one‐to‐one interviews, while members of the research team (E.E.J., E.B., D.S., B.H. and F.P.) facilitated the focus groups. We did not carry out repeat interviews or focus groups.

### 2.6 Data Analysis

Once complete, we downloaded recordings and transcripts from either Zoom or Microsoft Teams and stored these securely on Microsoft Teams; these were only available to the research team. One researcher (E.E.J.) fully anonymised all transcripts and reviewed the recordings, amending transcripts as necessary for typos, inaccuracies and formatting. For focus groups, one researcher (E.E.J.) also downloaded and fully anonymised the chat log from Zoom for analysis. We did not return transcripts to participants for comment or correction and did not discuss data saturation.

We undertook a framework analysis against Version 2 of the Theoretical Domains Framework (TDF) to enable comparison between the interviews and focus groups. One researcher (E.E.J.) created a framework in Microsoft Excel to deductively code against the TDF [13]. We used the TDF as a framework twice: once for coding barriers to public involvement and once for coding facilitators. Once each focus group and interview was fully coded against the TDF, one researcher (E.E.J.) carried out triangulation of analysis, using thematic analysis as described by Braun and Clarke to inductively code emerging themes within the data [14]. This was an iterative process, whereby themes were consistently reviewed and emergent themes were added or clustered as needed.

We counted the number of times a theme was identified against each domain of the TDF for each focus group and interview, which was quantified and tabulated in a separate Excel spreadsheet for both barriers and facilitators. To promote easier knowledge translation and categorisation of behaviours and themes, we further clustered the TDF domains into the three domains of the COM‐B behaviour change model using the structure suggested by Atkins et al [13]. Finally, the number of times each theme emerged from each domain of the COM‐B was tabulated and used to generate four tree maps visualising the barriers and facilitators identified in both the focus groups and interviews. Within the tree diagrams, the size of the sections and squares represented the frequency of each COM‐B domain and theme. We then narratively described the results of these analyses, using indicative quotations from both the focus groups and interviews to illustrate each point.

We invited eight participants from focus groups with members of the public to an engagement event to hear about the findings of the research and to give their opinions on the findings and next steps.

### 2.7 Reflexivity

Throughout the focus group and interview design and delivery process, E.E.J. maintained a reflexive diary based on the following concepts outlined by Olmos‐Vega et al. [15]: personal; interpersonal; methodological; and contextual. After each interview and focus group, E.E.J. drafted field notes and reflections based on the four concepts. The reflexivity process allowed E.E.J. to reflect on how the concepts may have potentially impacted on the overall conduct of each focus group and interview. In particular, the semistructured topic guide for the interviews was restructured and further defined by the reflexive process.

Reflexivity according to the Olmos‐Vega et al. principles was also exercised by E.E.J. in relation to data coding and analysis. In doing so, E.E.J. was able to further reflect on the decisions made during the analytical phase of the project and how this may potentially impact on the overall results.

### 2.8 Reporting

We have conducted reporting of this work in line with the COREQ Checklist for qualitative research (see Supplementary Material S6) [16].

## 3 Results

### 3.1 Characteristics of Participants

Twenty‐six members of the public joined the four focus groups. Details of the demographics of each focus group and answers to two Zoom polls conducted as part of the focus groups are shown in Table 2.

**Table 2:** Characteristics of focus group participants.

	Total (*n* = 26)	Group 1 (*n* = 8)	Group 2 (*n* = 6)	Group 3 (*n* = 6)	Group 4 (*n* = 6)
Age range					
18–29	5	4	1	0	0
30–49	9	3	1	3	2
50–64	6	0	3	1	2
65 and over	6	1	1	2	2
Prefer not to say	0	0	0	0	0
Geographic location in the UK					
North East	9	3	2	3	1
North West	2	0	0	0	2
Midlands	4	0	2	1	1
South West	3	3	0	0	0
London	5	1	1	2	1
South East	1	0	1	0	0
Scotland	0	0	0	0	0
Wales	0	0	0	0	0
Northern Ireland	1	0	0	0	1
Prefer not to say	1	1	0	0	0
Ethnic group					
White	13	3	3	3	4
Asian/Asian British	7	1	2	2	2
Black/African/Caribbean/Black British	4	3	0	1	0
Chinese	0	0	0	0	0
Arab	1	1	0	0	0
Other ethnic group	1	0	1	0	0
Prefer not to say	0	0	0	0	0
Before signing up to this focus group, had you heard of NICE?					
Yes	21	5^a^	6	5^b^	5^b^
No	2	2^a^	0	0^b^	0^b^
Before signing up to this focus group, had you heard of NICE's Technology Appraisal process?					
Yes	12	3^a^	3	2^b^	4^b^
No	11	4^a^	3	3^b^	1^b^

Abbreviation: NICE, National Institute for Health and Care Excellence.

^a^ Eight participants attended focus group 1 but only seven were present for the poll.

^b^ Six participants attended focus groups 3 and 4 but only five completed the poll.

Ten researchers involved in EAGs participated in one‐to‐one interviews. Characteristics of the researchers interviewed are shown in Table 3.

**Table 3:** Demographics of researchers involved in one‐to‐one interviews.

	*N* Interviewees (*n* = 10)
Number of EAG reports involved in	
0	0
1–10	4
11–20	2
21–30	2
31–40	1
More than 40	1
Role on EAG reports	
Clinical effectiveness reviewer	2
Health economics reviewer	1
Information specialist	0
Clinical effectiveness reviewer and overall lead	5
Health economics reviewer and overall lead	2
Previous experience of embedding PPIE into EAG reports	
Yes	2
No	8

Abbreviations: EAG, Evidence Assessment Group; PPIE, Patient and Public Involvement and Engagement.

### 3.2 Barriers

**Figure 1:**
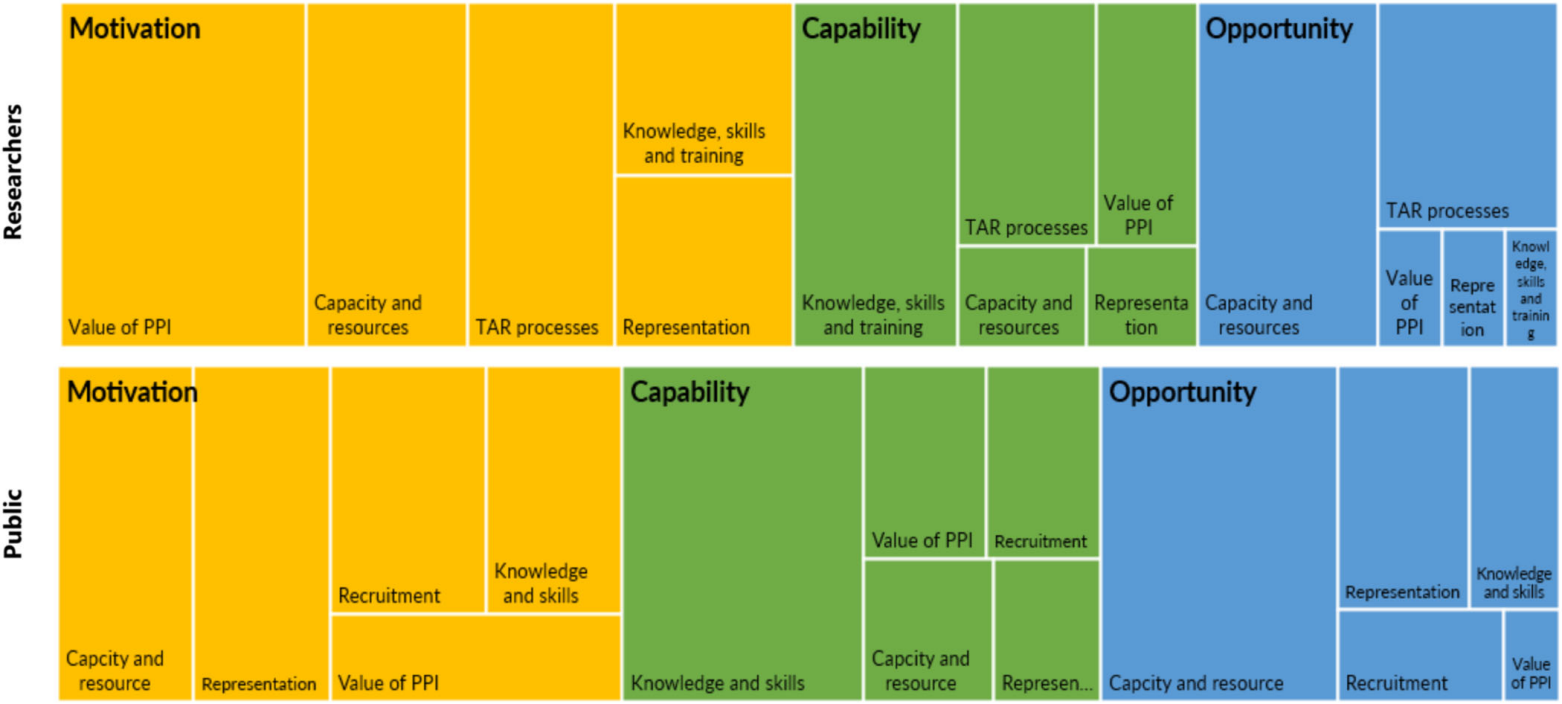
Tree charts comparing barriers to PPI for researchers and the public.

Tree diagrams of the barriers identified by the interviews and focus groups are displayed and compared in Figure 1.

#### 3.2.1 Potential Lack of Value of PPI in EAG Reports

For researchers, the biggest barrier to embedding patient and public perspectives into EAG Reports was a lack of motivation stemming from the perception that there is potentially limited value surrounding PPI within their reports. Specifically, some researchers were unsure of what added value PPI would bring to the process of writing an EAG Report. They noted that patients and the public were already involved in the wider TA process, suggesting that adding PPI would not change their conclusions.

‘But I think a big question that I have and a lot of my colleagues have is trying to see what the added value would be in relation to the effort involved. Because there's already patient and public involvement at the committee’.—Researcher 1.

‘Why are we involving them if we're not going to change anything? So I, I can't see the value.’—Researcher 3.

Researchers sometimes had the concern that the lived experience (or intrinsic knowledge) of patients and the public may not be relevant to the specific purpose of their reports; this was also tied to a perceived lack of other forms of knowledge and understanding that could prohibit involvement in reports. However, other researchers suggested that there would be additional benefit in embedding PPI into their reports, particularly in terms of understanding specific conditions or care pathways.

‘But that I think in terms of, you know, it's it might help you understand sometimes the reality of patient pathways and the, and it might help understand how important outcome is’—Researcher 8.

Members of the public rarely suggested that the value of PPI to an EAG Report was a barrier. When some members of the public did question the value of PPI in EAG Reports, they spoke about it in terms of having a lack of feedback on their involvement and how it had shaped or influenced work. Despite this, some still acknowledged that the need to add value through embedding patients and the public into EAG Reports was necessary but could prove challenging.

‘I think the challenge for you is. How can you add value to the process by having patients involved?‘—Member of the public, Group 4.

#### 3.2.2 Lack of Time, Capacity and Resource to Conduct PPI

For both researchers and members of the public, the biggest barrier to the opportunity to be able to conduct PPI in EAG Reports was a lack of capacity and resource to do so. Both groups suggested that a lack of time was a major barrier to involvement. For researchers, their perceived lack of capacity was often intrinsically linked to the short timelines within the current TA process and the challenges associated with this.

‘I really think that it would be very challenging to do it in current timelines and time pressures to do that as an additional thing as we're doing now.’—Researcher 8.

Members of the public agreed that time pressures faced by researchers in EAG Reports were difficult to surmount but also highlighted that people looking to be involved have time and resource barriers, including financial barriers, due to personal circumstances. This was particularly evident when reflecting on the vignette about ‘Lisa’ and potential barriers to her participation in a fictional EAG Report surrounding a new inhaler for asthma.

‘she may not be able to afford to get involved. Um. Unless she can pay for some childcare.’—Member of the public, Group 4.

By contrast, in the specific context of diagnostic accuracy reviews (DARs), one researcher contradicted the notion that the timeframe to undertake EAG reports was prohibitive to PPI but, aligning with other interviewees, noted that further human resources and capacity were a barrier to involvement.

‘I mean, I don't think timeline is is is the real issue timelines is probably, timelines are probably OK. It's more resources that you need to have extra time to engage’.—Researcher 5.

#### 3.2.3 Lack of Knowledge and Skills to Undertake PPI in EAG Reports

Researchers and members of the public both suggested that a lack of knowledge and skills across several areas prevented PPI in EAG reports. Members of the public expressed concerns surrounding the recruitment process, particularly that not knowing about opportunities to be involved was prohibitive. One member of the public highlighted that ‘people from different background are not fully aware of this, er, patient public engagement that is happening’, (Group 1) suggesting that social and cultural diversity and representation in PPI may also be prohibited by a lack of knowledge surrounding processes.

One of the largest knowledge and skills barriers prohibiting involvement for both researchers and members of the public was the technical detail within EAG Reports.

‘It's very very technical and it requires people with specific. Um. Credentials. To just do it as quick as possible and as precise as possible.’—Researcher 3.

‘Will the reports be in plain English? Because if they are very technical, people will get, like me, will get lost’—Member of the public, Group 3.

Researchers often saw the benefits of writing in plain language but expressed that rewriting technical language and jargon could be challenging, time consuming and potentially require additional training. Tied to this, some researchers felt that PPI skills within EAGs may potentially be lacking, which would prohibit their ability to involve patients and the public in their Reports quickly and confidently.

### 3.3 Facilitators

**Figure 2:**
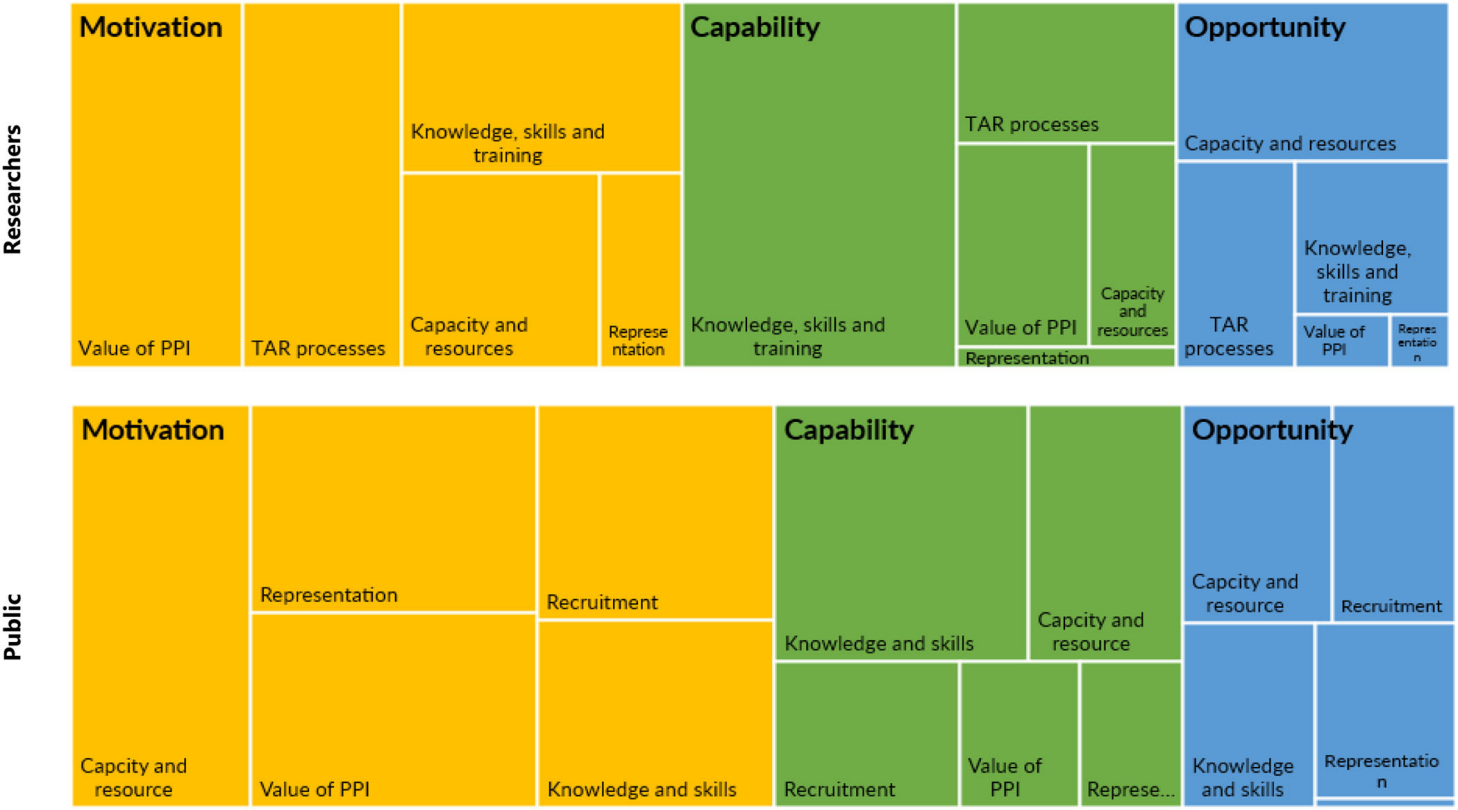
Tree chart comparing facilitators to PPI between researchers and the public.

Tree diagrams of the facilitators to PPI in EAG Reports identified by the interviews and focus groups are displayed and compared in Figure 2.

#### 3.3.1 Increasing Understanding of How PPI Can Be Embedded Into EAG Reports

Both members of the public and researchers highlighted that further resources, particularly defined guidance surrounding how to do PPI within EAG Reports, would help increase opportunities for involvement.

‘I think having a PPI strategy is where it begins. So having a specific. Document or guideline or a toolkit really, to help people understand, their role and some things which I have learned over the years’—Member of the public, Group 3.

‘Guidance really is needed on how do we do this well. For researchers and for patients within these NICE reports.’—Researcher 9.

People involved in our engagement event also emphasised the need for a framework for involvement in EAG Reports, and that patients and members of the public should be included in creating these guidelines.

Members of the public agreed that limiting the amount of technical medical language in reports would aid their potential involvement, with some suggesting a ‘jargon dictionary’ (Group 1) or glossary as a resource. Plain language summaries in EAG Reports were highlighted as a way in which the barrier to entry to involvement in EAG Reports for members of the public could be mitigated; this was discussed in depth in both focus groups and interviews. However, it was also noted, particularly from researchers, that further guidance would help facilitate this.

#### 3.3.2 Sharing Knowledge and Drawing on Already‐Established Networks

Sometimes the motivation to embed PPI into EAG Reports stemmed from knowing that capacity and resource would be available to do so. To mitigate the lack of time, capacity and resource potentially inhibiting PPI in EAG Reports, members of the public and researchers suggested ways in which the resource burden on the EAG could be reduced. Some members of the public suggested drawing on already‐established community centres, charities and organisations such as the Research Support Service (RSS). Furthermore, both researchers and the public suggested that a dedicated pool of members willing to be asked for contributions at short notice could be established.

‘And I did wonder if having that kind of existing pool of people. That you're going to, you're going to sort of work with over time, there's more of a kind of. I don't want to say the word pay off, but, you know, I mean, like investing more in that, and giving training’.—Researcher 9.

‘I think the practicalities are come, come. Not only be overcome, but can be more readily overcome if you've got the panel already in place, because then you can already have briefed um the individuals on the panel, on what, you know, what's involved in general terms in a PPI review’—Member of the public, Group 2.

Both groups also highlighted that further collaboration with NICE would help EAGs identify people willing to be involved in individual EAG Reports, particularly those with lived experience.

‘Because NICE are already going out and finding these patients. So why double up a resource? Would be my thought.’—Researcher 4.

#### 3.3.3 Increasing Awareness of the Technology Appraisal Process

Members of the public highlighted the need for information regarding TAs and additional transparency in the PPI process. They suggested that: there needed to be more awareness of TAs and EAG Reports; members of the public potentially looking to be involved should be informed of what PPI within the context of EAG Reports would consist of; they should know what researchers would require of them as part of their role (i.e., defined expectations); and have greater understanding of the impact their input has had on the work.

‘It's great to get out into the community and engage with people—educate and advise on how to get involved, the benefits of it, and why it's so important. Where to find ads in the first place for PPI. If people aren't aware of it then they can't get involved’—Member of the public, Group 2 (chat log).

Further reflecting on this theme, attendees at our engagement event emphasised that it is difficult for people to be able to contribute to a process if they do not have enough knowledge or information about what it entails. They again reiterated that knowing more about the process would enable them to make more meaningful contributions.

## 4 Discussion

### 4.1 Summary of Main Findings

Overall, 26 members of the public joined four focus groups and 10 researchers involved in EAGs participated in the one‐to‐one interviews.

The key barrier preventing PPI in EAG Reports for researchers was a perceived lack of value surrounding involving patients and the public, including being unsure of the added value and whether patients' experiences could meaningfully impact on conclusions. For both members of the public and researchers, time, capacity and resource (such as having the financial ability to be involved), as well as having the knowledge and skills to be able to be part of the process or undertake PPI effectively were also key barriers.

In terms of facilitators, both researchers and members of the public highlighted the need for defined guidance surrounding how to involve people within EAG Reports, as well as guidelines on how to write plain language summaries and reduce the amount of technical language within reports. Drawing on already‐established community centres, charities and organisations could be a way of identifying suitable people to be involved within EAG Reports, while members of the public expressed they would like to know more about what the report‐writing process consisted of, what would be required from them if they were involved and what impact their involvement has had on the work.

### 4.2 Implications for Practice and Future Research

In the short term, engagement with patients and members of the public could help raise awareness of what TAs are, how they impact on the NHS and how patients and the public feed into the process (e.g., through community groups). Both researchers and members of the public highlighted a lack of skills and knowledge that impacted on their capability to undertake PPI in the context of EAG Reports. Researchers could be signposted to resources, such as the NIHR's Learning for Involvement page, while the GET‐IT Glossary offers plain language translations of many terms related to health research, including health economics, which could help researchers translate technical terms into more easily understandable language. Researchers expressed they would like to see good practice examples of how PPI has been embedded into EAG Reports. Some examples of how PPI have shaped Early Value Assessments and DARs already exist and could be used to showcase potential methods and impact of PPI [17, 18].

In the longer term, EAG researchers and members of the public could assist in co‐creating guidance on how, and when, PPI can be meaningfully embedded into EAG Reports. As previously highlighted, some good practice examples of how PPI has shaped Early Value Assessments and DARs already exist [17, 18]. However, by further embedding PPI into different reports using a framework, the value of PPI within EAG Reports may become more evident. Furthermore, there is currently no specific guidance on how plain language summaries can be produced for EAG Reports. As highlighted by both researchers and members of the public, technical language and the use of acronyms and jargon is a barrier to involvement within EAG Reports. Further guidance would help EAGs in developing plain language summaries in their reports, thus increasing comprehensibility and usability for the public.

However, any such frameworks should be mindful of the time, capacity and labour in which PPI is undertaken within EAG Reports. As noted by previous research, PPI can require significant administrative labour [19]; this may not always be possible within the structural constraints of the EAG Report process, where an STA may take as little as 6 to 8 weeks to complete [20]. Indeed, aligning with previous research surrounding PPI in evidence synthesis [21], time and capacity was a noted barrier for participants within this research. It may be that what constitutes meaningful PPI in EAG Reports varies by the type of Report being produced (e.g., an STA as opposed to a DAR, where timeframes are longer, as noted by one researcher in this work). Notably, two examples of implementing PPI within EAG Reports are both embedded within Early Value Assessments and DARs, whereby the EAG are responsible for the collation and synthesis of the evidence [17, 18].

Furthermore, it has been noted that PPI within health economics modelling is a relatively recent development [22, 23], and that the language of health economic evaluation can be complex and inaccessible [24]. This reflects the technical detail of EAG Reports noted by researchers in this work. Although the development of the Consolidated Health Economic Evaluation Reporting Standards (CHEERS) 2022 checklist now contains two items regarding PPI in the development of health economic evaluations [25], it is still unclear precisely how this can be achieved, particularly in the specific, time‐constrained context of EAG Reports.

Reflecting on these points, more work could be undertaken to explore what constitutes meaningful involvement, how we can avoid potential tokenism, and demonstrate the potential value of PPI within different EAG Reports. The use of any frameworks to conduct PPI within EAG Reports should also evaluate the impact of PPI on the work from the perspective of both researchers and the public. Such evaluation should be a critical reflection that also considers the potential negative impacts of PPI within EAG Reports, to avoid involvement becoming a ‘tick box’ exercise [26].

### 4.3 Strengths and Limitations of This Study

The key strength of this work was the continual active involvement of two members of the public in the research team (D.S. and B.H.) in line with the UK Standards for Public Involvement [6, 7]. They co‐designed aspects of the focus groups, co‐facilitated focus groups and aided in decision‐making and interpretation of results. In all, 26 people attended the focus groups and the mix of people who had and had not heard of the TA process allowed us to capture a range of views. Ten researchers were involved in one‐to‐one interviews, again with a range of experience in how many EAG Reports they had previously been involved in. Two of the 10 researchers also had previous experience of embedding PPI into EAG Reports, offering another viewpoint.

However, there were limitations in the representativeness of participants within the study. Although those attending focus groups were purposively sampled to try and ensure a breadth of views were represented, most participants lived in the North East of England and were white. Although we used vignettes as a method for encouraging discussion surrounding a potentially unfamiliar topic area, it is also possible that participants in the focus groups may have reflected more on the barriers and facilitators presented within these vignettes rather than their own perspectives. Though we attempted to only use the vignettes as a starting point for wider discussion, this could potentially have introduced bias into the results. Additionally, EAG researchers were mainly clinical effectiveness reviewers (often with overall responsibility for the EAG Reports) and there was no representation from Information Specialists.

This study was funded by a career development grant intended to enhance research skills and capacity. In this context, this was the first time the lead researcher (E.E.J.) had led a primary qualitative study from inception to completion, though had previous experience in facilitating focus groups, workshops and qualitative analysis and was guided by experienced members of the research team and received advice from colleagues with extensive experience in qualitative research. This single researcher coded and analysed focus groups and interviews due to time and capacity limitations. Although one interview was coded by another researcher for quality assurance and E.E.J. critically reflected on their practice using principles outlined by Olmos‐Vega et al. [15], this may have introduced some bias into the findings. Furthermore, we did not discuss what constituted data saturation as a research team. This means it is possible that data collection may have ended before data saturation was reached and further emerging themes may have been missed.

## 5 Conclusion

This qualitative study including focus groups with members of the public and interviews with EAG researchers highlights key barriers and facilitators to PPI in EAG Reports feeding into NICE's TA process. Key recommendations from the work include developing a PPI framework for EAG Reports, the need for defined guidance on how to write plain language summaries within EAG Reports, and a need for good practice examples of PPI to demonstrate the value of involvement within EAG Reports.

## Author Contributions

**Eugenie Evelynne Johnson:** conceptualization, funding acquisition, investigation, methodology, project administration, data curation, formal analysis, validation, visualization, writing–original draft. **Debbie Smith:** methodology, validation, writing–review and editing. **Becky Harmston:** methodology, writing–review and editing, validation. **Emily Hunter:** data curation, writing–review and editing. **Emma Belilios:** conceptualization, funding acquisition, methodology, validation, supervision, writing–review and editing. **Fiona Pearson:** conceptualization, funding acquisition, methodology, validation, supervision, writing–review and editing.

## Consent

Participants in this qualitative study provided informed consent to take part in the focus groups and interviews.

## Conflicts of Interest

Eugenie Evelynne Johnson works as a Clinical Effectiveness Reviewer for the Evidence Assessment Group based at Newcastle University, UK. Emma Belilios works within the External Assessment Group based at Newcastle upon Tyne Hospitals NHS Foundation Trust, UK. Fiona Pearson is a co‐applicant for the Technology Appraisal Group at Newcastle University, UK and works as a Clinical Effectiveness Reviewer for both this Group and the External Assessment Group based at Newcastle upon Tyne Hospitals NHS Foundation Trust, UK. The remaining authors declare no conflicts of interest.

## Supporting information

Supporting information.

## Data Availability

Data are available from the authors upon reasonable request.

## References

[1] National Institute for Health and Care Excellence , *Technology Appraisal Processes* (National Institute for Health and Care Excellence), https://www.nice.org.uk/about/what-we-do/our-programmes/nice-guidance/nice-technology-appraisal-guidance/process.

[2] National Institute for Health and Care Research , *Briefing Notes for Researchers—Public Involvement in NHS, Health and Social Care Research* (National Institute for Health and Care Research 2021), https://www.nihr.ac.uk/documents/briefing-notes-for-researchers-public-involvement-in-nhs-health-and-social-care-research/27371.

[3] National Institute for Health and Care Excellence , *Involvement and Participation* (National Institute for Health and Care Research). Updated 2023, https://www.nice.org.uk/process/pmg36/chapter/involvement-and-participation.

[4] National Institute for Health and Care Research , *INCLUDE: Resources* (National Institute for Health and Care Research), https://sites.google.com/nihr.ac.uk/include/home/resources.

[5] A. Pollock , P. Campbell , C. Struthers , et al., “Development of the ACTIVE Framework to Describe Stakeholder Involvement in Systematic Reviews,” Journal of Health Services Research & Policy 24, no. 4 (2019): 245–255. 30997859 10.1177/1355819619841647

[6] UK Standards for Public Involvement , https://sites.google.com/nihr.ac.uk/pi-standards/home.

[7] S. Crowe , A. Adebajo , H. Esmael , et al., “‘All Hands‐On Deck’, Working Together to Develop UK Standards for Public Involvement in Research,” Research Involvement and Engagement 53 (2020): 1–6. 10.1186/s40900-020-00229-y PMC7493420 32974049

[8] Cardiff University , *Public Involvement in Research Impact Toolkit (PIRIT)* (Cardiff University), https://www.cardiff.ac.uk/marie-curie-research-centre/patient-and-public-involvement/public-involvement-in-research-impact-toolkit-pirit.

[9] M. Sandelowski , “Whatever Happened to Qualitative Description?,” Research in Nursing & Health 23 (2000): 334–340. 10940958 10.1002/1098-240x(200008)23:4<334::aid-nur9>3.0.co;2-g

[10] Qualtrics. Qualtrics. Provo, Utah (USA: Qualtrics, 2020) .

[11] National Institute for Health and Care Research , *Evidence Synthesis* (National Institute for Health and Care Research), https://www.nihr.ac.uk/explore-nihr/funding-programmes/evidence-synthesis.htm#five.

[12] H. Kara , Creative Research Methods: A Practical Guide (Policy Press, 2020).

[13] L. Atkins , J. Francis , R. Islam , et al., “A Guide to Using the Theoretical Domains Framework of Behaviour Change to Investigate Implementation Problems,” Implementation Science 12, no. 1 (2017): 77. 28637486 10.1186/s13012-017-0605-9 PMC5480145

[14] V. Braun and V. Clarke , “Using Thematic Analysis in Psychology,” Qualitative Research in Psychology 3, no. 2 (2006): 77–101.

[15] F. M. Olmos‐Vega , R. E. Stalmeijer , L. Varpio , and R. Kahlke , “A Practical Guide to Reflexivity in Qualitative Research: Amee Guide No. 149,” Medical Teacher 45, no. 3 (2022): 241–251. 10.1080/0142159X.2022.2057287 35389310

[16] A. Tong , P. Sainsbury , and J. Craig , “Consolidated Criteria for Reporting Qualitative Research (COREQ): A 32‐Item Checklist for Interviews and Focus Groups,” International Journal for Quality in Health Care 19, no. 6 (2007): 349–357. 17872937 10.1093/intqhc/mzm042

[17] H. Shabaninejad , R. P. W. Kenny , T. Robinson , et al., “Early Value Assessment: Genedrive MT‐RNR1 ID Kit for Detecting Single Nucleotide Polymorphism M,” 1555A>G in Neonates (Newcastle Technology Assessment Group, 2022). 10.3310/TGAC4201 PMC11590116 39487741

[18] E. Tomlinson , M. Ward , C. Cooper , et al., Point of Care Tests for Urinary Tract Infections (UTI) to Reduce Antimicrobial Resistance: A Systematic Review and Conceptual Economic Model to Inform Early Value Assessment (EVA) (DAP 69) (Bristol Technology Assessment Group, 2023). 10.3310/PTMV8524 PMC11647557 39644102

[19] A.‐M. Boylan , L. Locock , R. Thomson , and S. Staniszewska , “About Sixty Per Cent I Want to Do It”: Health Researchers’ Attitudes to, and Experiences of, Patient and Public Involvement (PPI)—A Qualitative Interview Study,” Health Expectations: An International Journal of Public Participation in Health Care and Health Policy 22, no. 4 (2019): 721–730. 30927334 10.1111/hex.12883 PMC6737750

[20] National Institute for Health and Care Excellence , *Single Technology Appraisal (STA) Timeline* (National Institute for Health and Care Excellence), https://www.nice.org.uk/about/what-we-do/our-programmes/nice-guidance/technology-appraisal-guidance/process/sta-timeline.

[21] E. Agyei‐Manu , N. Atkins , B. Lee , et al., “The Benefits, Challenges, and Best Practice for Patient and Public Involvement in Evidence Synthesis: A Systematic Review and Thematic Synthesis,” Health Expectations 26, no. 4 (2023): 1436–1452. 37260191 10.1111/hex.13787 PMC10349234

[22] S. Harvard and E. Winsberg , “Patient and Public Involvement in Health Economics Modelling Raises the Need for Normative Guidance,” PharmacoEconomics 41 (2023): 733–740. 37106229 10.1007/s40273-023-01274-7

[23] S. Staniszewska , E. M. Hill , R. Grant , et al., “Developing a Framework for Public Involvement in Mathematical and Economic Modelling: Bringing New Dynamism to Vaccination Policy Recommendations,” The Patient Patient‐Centered Outcomes Research 14 (2021): 435–445. 33462773 10.1007/s40271-020-00476-x PMC8205902

[24] S. Staniszewska , I. Jakab , E. Low , et al., “Commentary: Advocating for Patient and Public Involvement and Engagement in Health Economic Evaluation,” Research Involvement and Engagement 9, no. 45 (2023): 45. 37400923 10.1186/s40900-023-00444-3 PMC10316557

[25] D. Husreau , M. Drummond , F. Augustovski , et al., “Consolidated Health Economic Evaluation Reporting Standards 2022 (CHEERS 2022) Statement: Updated Reporting Guidance for Health Economic Evaluations,” MDM Policy & Practice 7, no. 1 (2022): 23814683211061097. 35036563 10.1177/23814683211061097 PMC8755935

[26] J. Russell , N. Fudge , and T. Greenhalgh , “The Impact of Public Involvement in Health Research: What Are We Measuring? Why Are We Measuring It? Should We Stop Measuring It?,” Research Involvement and Engagement 6, no. 63 (2020): 63. 33133636 10.1186/s40900-020-00239-w PMC7592364

