# Supplementary material for: Exploring Barriers and Facilitators to Patients and Members of the Public Contributing to Rapid Health Technology Assessments for NICE: A Qualitative Study
Source: Health Expect. 2024 Nov 18;27(6):e70109. doi: 10.1111/hex.70109 (PMC11573722; doi:10.1111/hex.70109)
Supplement: Supplementary file 1 — Supporting information. [file HEX-27-e70109-s001.docx]

**Supplementary Material 1: Information sheet and consent form for public focus groups**

**Help us with our research to understand more about how patients and the public can be involved in reports contributing to NICE’s Technology Appraisal process**

 **Who are we looking for?**
 Are you interested in how patients and the public can be involved in reports that feed into decisions made by the National Institute for Health and Care Excellence (NICE) on what health technologies are approved for use in the NHS? If so, we invite you to take part in our research to help us identify what is preventing people being involved in these reports and how we can enable them to take part.

 Before deciding to take part, it is important you understand why the research is being done and what it will involve. Please read the following carefully and discuss it with others if you wish.

 If anything is not clear or you would like more information, please email Eugenie Johnson at: eugenie.johnson@newcastle.ac.uk

 **Why are we doing this study?**
 The National Institute for Health and Care Excellence (NICE) produces national guidance on health technologies for the NHS. Health technologies can be medicines, devices or digital interventions. NICE commissions independent research groups to carry out thorough assessments of new medicines or medical devices, including digital devices, for a particular health condition. These groups look at the evidence on how effective the health technology is, how safe it is, and whether it represents value for money to the NHS. The reports they produce go to NICE Committees, who make recommendations on whether the health technology can be used in the NHS. The groups talk to companies and clinical experts when they are producing their reports, but there is little or no input from people with lived experience of the relevant health condition.

We want to find out how best to involve people with lived experience in developing the independent reports in a structured and consistent way, to strengthen national guidance and improve healthcare. It will explore barriers that may discourage people from taking part and how these may be overcome. To do this, we will be conducting focus groups with members of the public and one-to-one interviews with researchers involved in the independent reports.

We are hoping to share the findings as an academic paper and as an accessible graphic for the public.

 **What will taking part in the study involve?**
 An English language focus group with up to nine other people, conducted and recorded on Zoom or Microsoft Teams.

 You will be given a short presentation on what NICE Technology Appraisals are and on Technology Appraisal Reports. We will then ask the focus groups to discuss what might prevent people from becoming involved in these reports and what could enable them to engage. We will not ask any personally-identifying information during the focus group. For your time, you will receive a £25 digital gift voucher.

 This study has received ethics approval from Newcastle University Ethics Committee.

 **What data will we collect and store?**
 We will ask you to provide your name as an indication of consent to take part and your e-mail to allow us to contact you and help to facilitate payment of a voucher in recognition of your time. We will keep this data separate to any other data collected.

 We will briefly ask all participants in the focus group to give an indication of whether they have previously participated in research using a poll but no other personally identifying information will be gathered during the focus group.

 We will record focus groups to allow for accurate capture of discussions. Recordings will be typed up by the main researcher on this project, anonymised, then deleted. Data collected will be stored securely using a Newcastle University encrypted platform which only the researchers involved in the project will have access to. We will only disclose information you provide if there is a legal requirement to do so (for example, under statute or a court order) and/or we have an overriding duty to the public (for example, the information concerns the commission of a criminal offence or relates to life-threatening circumstances).

 Newcastle University will act as the data controller for this study, ensuring your information is used appropriately and in the public interest. You can find out more about this here https://www.ncl.ac.uk/data.protection/dataprotectionpolicy/privacynotice/ or by contacting Newcastle University's Data Protection Officer (Maureen Wilkinson, rec-man@ncl.ac.uk).

 **Are there any risks in taking part?**
 Taking part in this project will lead to a better understanding of how patients and the public can be involved in the report process and poses limited risks to you. We will not be discussing any sensitive issues. However, if you do experience distress during the focus group, you can pause or stop your participation as needed, or you may withdraw from the focus group.

 **Can I stop taking part?**
 If you do decide to take part, you can stop at any point without a reason just by letting the researcher know. If you stop after the recording of the focus group has been transcribed or anonymised, your consent form and email address will be deleted but we will be unable to identify your interview data for removal.

 **Who do I contact if I have a concern about the research, or I wish to complain?**
 If you have a concern or any queries about any aspect of this study, please contact Miss Eugenie Johnson: eugenie.johnson@newcastle.ac.uk

End of Block: Default Question Block

Start of Block: Block 1

Q2 Are you over 18 years old?

- Yes (1)
- No (2)

End of Block: Block 1

Start of Block: Block 2

Q3 Please complete the following consent form while considering the information you received previously and/or discussed afterwards.

Q4 I confirm that I have read the information sheet provided, had the opportunity to consider the information, had the opportunity to ask questions and have had any questions answered satisfactorily.

- Yes (1)
- No (2)

Q5 I understand my participation is voluntary and that I can stop at any time. I understand that if I stop, any data I have provided up to that point (as far as is plausible) will be deleted.

- Yes (1)
- No (2)

Q6 I understand how my personal information will be used for this study.

- Yes (1)
- No (2)

Q7 I understand that I will be audio recorded, that this will be stored anonymously on password-protected software, used for research purposes only then destroyed after completion of the transcription.

- Yes (1)
- No (2)

Q8 I understand that personal details, such as my name and email address, will not be revealed to anyone outside of the project.

- Yes (1)
- No (2)

Q9 I am happy for my anonymised, transcribed data to be stored and used by others for future research.

- Yes (1)
- No (2)

Q10 I am happy for my e-mail address to be stored for up to 12 weeks so I can receive a gift voucher upon study completion.

- Yes (1)
- No (2)

End of Block: Block 2

Start of Block: Block 3

Q11 Please confirm that you agree (consent) to take part in this research project by providing the information below.

Q12 Your name

________________________________________________________________

Q13 Your email address

________________________________________________________________

Q14 The date you completed this form

________________________________________________________________

Q22 Please tell us which workshop date and time would be most suitable for you.

- Wednesday 8th November - 11am to 12pm (1)
- Thursday 16th November - 10am to 11am (2)
- Friday 24th November - 2pm to 3pm (3)

Q24 In case your first choice of date is not available, please tell us your second choice.

- Wednesday 8th November - 11am to 12pm (1)
- Thursday 16th November - 10am to 11am (2)
- Friday 24th November - 2pm to 3pm (3)

End of Block: Block 3

Start of Block: Block 5

Q18 What is your age range?

- 18 to 29 (1)
- 30 to 49 (2)
- 50 to 64 (3)
- 65 and over (4)
- Prefer not to say (5)

Q19 Where in the United Kingdom do you live?

- North East (1)
- North West (2)
- Midlands (3)
- South West (4)
- London (5)
- South East (6)
- Scotland (7)
- Wales (8)
- Northern Ireland (9)
- Prefer not to say (10)

Q20 What is your ethnic group?

- White (1)
- Asian/ Asian British (2)
- Black/ African/ Caribbean/ Black British (3)
- Chinese (4)
- Arab (5)
- Other ethnic group (6)
- Prefer not to say (7)

End of Block: Block 5

Start of Block: Block 4

Q15 Thank you for expressing your interest in joining one of our focus groups. 

Once we have our findings from the project, we will be organising a one-hour, online workshop to present these findings to people who have contributed as part of the focus groups and allow you to have the chance to ask questions or raise any additional points. We aim to have a live scribe attend the event to create a graphic of our findings and to change aspects of these findings in “real time”. This graphic will be used to help share our findings with other members of the public but you will not be identifiable.

The workshop will take place in early 2024 and your time attending the workshop will be recognised with a £25 digital voucher.

Q16 Would you be interested in joining this one-hour workshop?

- Yes (1)
- No (2)

End of Block: Block 4

**Supplementary Material 2: Information sheet and consent form for interviews with researchers**

Help us with our research to understand more about how patients and the public can be involved in reports contributing to Technology Appraisal Reports

 **Who are we looking for?**
 Do you have experience of working on NICE Technology Appraisal Evidence Assessment Group Reports as part of an Evidence Assessment Group? Have you involved patients or the public when you have conducted these Reports or have ideas on how we could involve patients and the public in future? If so, we invite you to take part in our research to help us identify what is preventing patients and the public being involved in these reports and how we can enable them to take part.

 Before deciding to take part, it is important you understand why the research is being done and what it will involve. Please read the following carefully and discuss it with others if you wish.

 If anything is not clear or you would like more information, please email Eugenie Johnson at: eugenie.johnson@newcastle.ac.uk

 **Why are we doing this study?**
 NICE Technology Appraisal include rapid critical appraisal of clinical- and cost-effectiveness evidence submitted by a company on a health technology by an independent Evidence Assessment Groups (EAGs). Historically, patient and public members do not contribute directly to the EAG’s report, although they do inform the Committee’s decision-making process. Patient and public involvement and engagement (PPIE) is an expectation of healthcare researchers. As Technology Appraisal reports are produced at pace, what constitutes meaningful PPIE within EAG reports is challenging, particularly as there is no framework outlining what is considered “good practice” in this context.

 In this research project, we want to find out what is preventing PPIE in these reports and how it can be facilitated, taking the perspectives of both researchers involved in Technology Appraisal Reports and those of patients and the public. To do this, we will be conducting focus groups with members of the public and one-to-one interviews with researchers involved in EAG Reports.

 We are hoping to share the findings as an academic paper and as an accessible graphic for the public.

 **What will taking part in the study involve?**
 A one-to-one, English language interview conducted and recorded on Zoom or Microsoft Teams. You will asked a series of questions about PPIE within EAG Reports, including what barriers and facilitators there may be to involving patients and the public in the process from your perspective. This study has received ethics approval from Newcastle University Ethics Committee. What data will we collect and store? We will ask you to provide your name as an indication of consent to take part. We will keep this data separate to any other data collected. At the start of the interview, you will asked for your job title and an approximation of how many Technology Appraisal EAG Reports you have been involved in writing. This data will be used to gauge whether the participants mainly work on the clinical- or cost-effectiveness component and the level of experience in EAG Reports the interview participants have.

 We will record interviews to allow for accurate capture of discussions. Recordings will be transcribed by the main researcher on this project, anonymised, then deleted. Data collected will be stored securely using a Newcastle University encrypted platform which only the researchers involved in the project will have access to. We will only disclose information you provide if there is a legal requirement to do so (for example, under statute or a court order) and/or we have an overriding duty to the public (for example, the information concerns the commission of a criminal offence or relates to life-threatening circumstances).

 Newcastle University will act as the data controller for this study, ensuring your information is used appropriately and in the public interest. You can find out more about this here https://www.ncl.ac.uk/data.protection/dataprotectionpolicy/privacynotice/ or by contacting Newcastle University's Data Protection Officer (Maureen Wilkinson, rec-man@ncl.ac.uk).

 **Are there any risks in taking part?**
 Taking part in this project will lead to a better understanding of how patients and the public can be involved in the Technology Appraisal Report process and poses limited risks to you. We will not be discussing any sensitive issues. However, if you do experience distress during the interview, you can pause or stop your participation as needed, or you may withdraw from the interview.

 **Can I stop taking part?**
 If you do decide to take part, you can stop at any point without a reason just by letting the researcher know. If you stop after the recording of the interview has been transcribed or anonymised, your consent form will be deleted but we will be unable to identify your interview data for removal.

 **Who do I contact if I have a concern about the research, or I wish to complain?**
 If you have a concern or any queries about any aspect of this study, please contact Miss Eugenie Johnson: eugenie.johnson@newcastle.ac.uk

End of Block: Default Question Block

Start of Block: Block 1

Q3 Are you over 18 years old?

- Yes (1)
- No (2)

End of Block: Block 1

Start of Block: Block 2

Q4 **Consent form**

 Please complete the following consent form while considering the information you have read above and/or subsequently discussed.

Q5 I confirm that I have read the information sheet provided, had the opportunity to consider the information, had the opportunity to ask questions and have had any questions answered satisfactorily.

- Yes (1)
- No (2)

Q6 I understand my participation is voluntary and that I can stop at any time. I understand that if I stop, any data I have provided up to that point (as far as is plausible) will be deleted.

- Yes (1)
- No (2)

Q7 I understand how my personal information will be used for this study.

- Yes (1)
- No (2)

Q8 I understand that I will be audio recorded, that this will be stored anonymously on password-protected software, used for research purposes only then destroyed after completion of the transcription.

- Yes (1)
- No (2)

Q9 I understand that personal details, such as my name and email address, will not be revealed to anyone outside of the project.

- Yes (1)
- No (2)

Q10 I am happy for my anonymised, transcribed data to be stored and used by others for future research.

- Yes (1)
- No (2)

Q11 I understand and agree my data may be published as a journal article and may appear in other materials used to share the findings of the project.

- Yes (1)
- No (2)

Q12 I am happy for my anonymised, transcribed data to be stored and used by others for future research.

- Yes (1)
- No (2)

End of Block: Block 2

Start of Block: Block 3

Q14 Please confirm that you agree (consent) to take part in this research project by signing below.

Q15 Your name

________________________________________________________________

Q16 Your email address

________________________________________________________________

Q18 Date you completed this consent form

________________________________________________________________

End of Block: Block 3

**Supplementary Material 3: Semi-structured topic guide for interviews with researchers**

**Interviewer name**:

**Participant number**:

**Interview date**:

**Interview topic guide**

| **Introduction** | Thank you for agreeing to take part in this interview. As noted on the information sheet, the purpose of this interview is to find out more about the perspectives of researchers involved in Evidence or External Assessment Groups feeding into NICE’s Technology Appraisal process on patient and public involvement in their reports.  We are doing this as part of a preliminary investigation into barriers and facilitators to patient and public involvement in EAG Reports and, alongside interviewing researchers about their views, we are also going to be doing a series of focus groups with the public to gain their perspectives as well.  Participation in this interview is voluntary. Everything that we discuss during this interview will be kept confidential and only shared with members of the research team anonymously. However, if I feel there is potential for someone to be at risk of harm then confidentiality may need to be broken.  Do you have any questions about what was provided to you on the information sheet regarding the project or your participation?  Do you still consent to take part in this interview?  Are you happy for the audio and video of this interview to be recorded and transcribed?  Thank you, I will now begin the recording of this interview. |
| --- | --- |
| **If still consenting and all prior queries answered, begin recording here** | |
| **Background queries** | - To test that the recording and Teams transcription is working correctly, can you please confirm your name? Your name will be anonymised and not included in any reports or publications. - To start the collected demographic information, approximately how many EAG Reports have you worked on in the past? - Do you usually work as a clinical effectiveness reviewer, a health economic reviewer, an information specialist or an overall project lead on EAG Reports? - How do you see your role when you are working on EAG Reports and Technology Assessments? (Potential prompt: In brief, what types of tasks do you tend to perform?) - Do you have any prior experience of embedding patient and public involvement into EAG Reports? (yes/no) |
| **Q1** | Can you tell me a bit about:   - If they have done PPIE previously: Can you tell me a bit more about **how you have** embedded PPIE into EAG Reports in the past?   - NB: Probe deeper into the facilitators here and pick up on any potential challenges if needed; head to Q2 - If they haven’t: **Would you consider** embedding PPIE into your EAG Reports in the future?   - NB: Depending on answer, either pick up on specific points and probe deeper or move on to Q2 |
| **Q2** | Looking back…  If they have embedded PPIE previously:   - Have there been any challenges when embedding patient and public involvement into EAG Reports?   If they have not embedded PPIE previously:   - What do you think would be the major challenges in embedding PPIE into EAG Reports?   - NB: If they have noted some challenges in Q1, ask more about these.   Potential further prompts if required:   - Capacity/resource - Time frames to completion - Confidentiality - Generalisability/representativeness of PPIE |
| **Q3** | Looking back…  If they have embedded PPIE previously:   - What has helped you to embed patient and public involvement into EAG Reports in the past?   If they have not embedded PPIE previously:   - What may potentially help you in the future to embed patient and public involvement into EAG Reports? |
| **Q4** | Thinking about internal systems, how might these need to be changed to help facilitate embedding patient and public involvement in EAG Reports? |
| **Q5** | Looking forward, what do you see as priority actions which could be carried out soon to help EAGs embed patient and public involvement in their reports? |
| **Q6** | If all constraints were removed and you could direct what is done, is there anything you would like to see included as standard in terms of patient and public involvement in EAG Reports? |
| **Q7** | Overall, do you think that it is important to embed patient and public involvement into EAG Reports? |
| **Close** | **Closing statement**  That’s the end of the questions I have, thank you so much for your time.  Once the analysis is done, I will develop a report with the findings and plan to produce an academic publication surrounding the findings as well. My intention is to keep all volunteers updated with progress and I would be happy to answer any questions you might have about the project going forward.  Are you happy to be contacted regarding the results of the project going forward?  Do you have any other questions?  Thank you for your time. |

**Supplementary Material 4: Semi-structured topic guide for focus groups with members of the public**

| **Focus groups with the public: Barriers and facilitators to PPIE in Technology Appraisal Reports**  **Dates:**   - 8^th^ November – 11:00-12:00 - 16^th^ November – 10:00-11:00 - 24^th^ November – 14:00-15:00   **Length:** 1 hour  **Location**: Zoom  **Facilitator:** Eugenie Johnson (any others TBC) | |
| --- | --- |
| **Timing** | **Activity** |
| 5 mins | **Introduction**  Hello everyone and welcome to this focus group exploring what might help or prevent patients and members of the public from being involved in reports that feed into the National Institute for Health and Care Excellence’s (NICE’s) Technology Appraisal process.  Before we begin, it’d be great just to get to know a little more about each other. So, I’m going to go around the Zoom room and ask everyone to say their name and tell us one word that describes how they’re feeling today. I’ll start off.  [Go around research team first and then go on to focus group participants]  [**Put in chat**: Introductions – let us know your name and tell us one word that describes how you’re feeling today]  Housekeeping - explanation about recording/confidentiality; any objections – chance for people to leave/withdraw consent |
| **Start recording here**  **Enable closed captioning if required** | |
| 5 mins | **Brief Zoom polls – Before joining this focus group...**   - Had you heard of NICE? (yes/no) - Had you heard of NICE Technology Appraisals? (yes/no) |
| 10 mins | **Brief summary on NICE Technology Appraisals are and the role of EAG Reports**   - Why we are here: to explore how patient and public involvement can be embedded into these reports – read from PowerPoint - Short opportunity for Q&A |
| 15 mins | **Vignette 1: Ben and Lisa (barriers)**   - Read out vignette about Ben and Lisa - Key question: What might prevent Lisa, or others, from becoming involved in Ben’s report?   - Follow-up Q 1: Ben only has a week to ask Lisa some questions about this inhaler. Do you think that short time-frame would make it harder for Lisa to be involved?   - Follow-up Q2: Ben also isn’t sure if he has any money to be able to give any recognition payments for Lisa’s time. Do you think this might stop her from getting involved? - Participant can speak, use chat as they feel comfortable to do so |
| 15 mins | **Vignette 2: Samira and David (enablers)**   - Read out vignette about Samira and David - Key question 1: How can Samira enable David to be involved?   - Follow-up Q1: David wasn’t sure if he had enough knowledge about the process to help. What can Samira do to help David know more about the process?   - Follow-up Q2: David isn’t sure if he can respond quickly enough to meet Samira’s deadlines (within 8 weeks). Is there anything that can be done to help David contribute within this short time-frame? - Key question 2: Is there anything Samira needs so she can help David be involved?   - Follow-up Q1: What information does Samira need to know from David so that she can help him be involved?   - Follow-up Q2: Are there any resources that Samira needs to help David be involved? (e.g. any plain language materials or a plain language overview of the process)? - Participant can speak, use chat as they feel comfortable to do so |
| **Stop recording here** | |
| 5 mins | **Final comments:**  Thank you very much for taking the time to talk to us today. In recognition of your time today we will be sending £25 of Gift Pay vouchers to you via email. This is a bit like Love2Shop vouchers but offers greater flexibility in retailers. We will be arranging that in the next couple of days.  In early 2024, we will be holding a meeting to present the results and get any further feedback from patients and members of the public. Unfortunately, only have capacity for 10 people to attend this event from all of the focus groups we are holding, so we can’t guarantee that you’ll be able to attend. However, we will be sending out a plain language version of the results to everyone who expressed an interest in attending the event so you will all be kept in the loop.  Does anyone have any final questions?  If you have any questions at all following this focus group, then please feel free to email me. You will have my email address from the invitation I sent out but I will also quickly put it in the chat. [Put email address in chat for reference]  Once again, many thanks for attending and I hope you enjoy the rest of your day.  Close |

**Supplementary Material 5: Vignettes used in the focus groups**

These vignettes were created using Canva Pro and Microsoft PowerPoint.


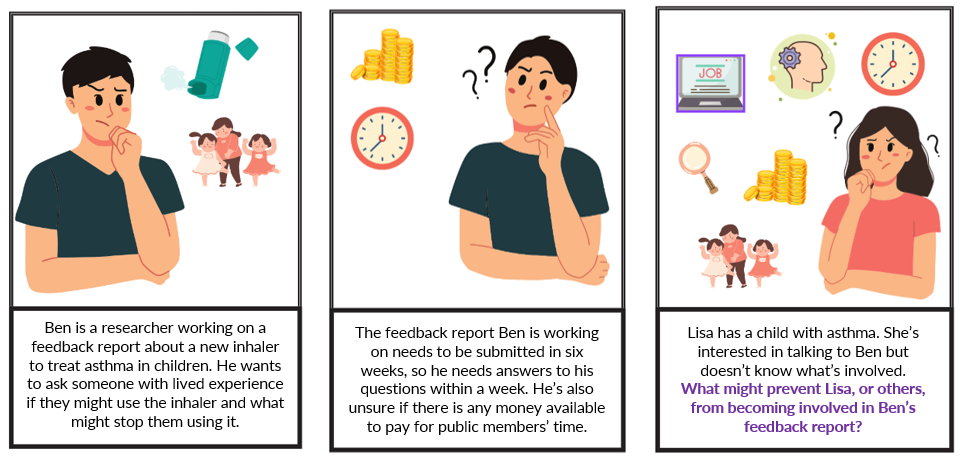


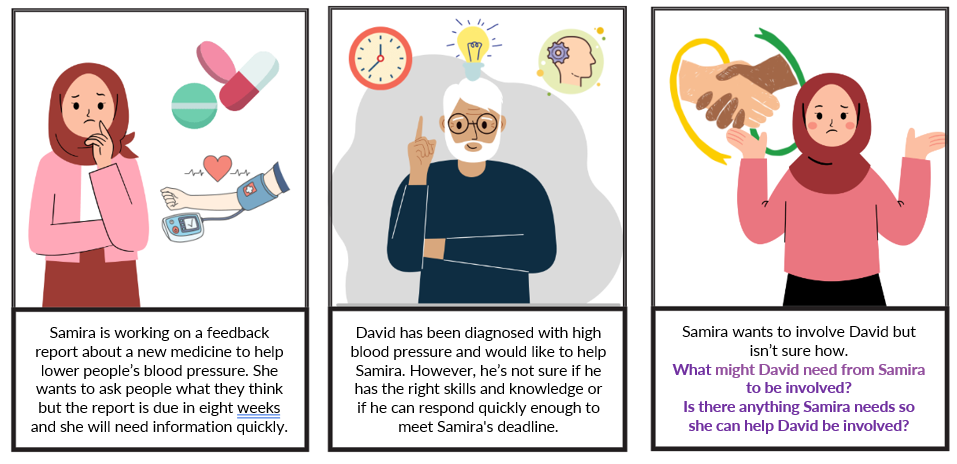


| **Supplementary Material 6: COREQ Checklist** | | | |
| --- | --- | --- | --- |
| **No** | **Item** | **Guide questions/description** | **Page reported** |
| **Domain 1: Research team and reflexivity** | | | |
| **Personal Characteristics** | | | |
| 1. | Interviewer/facilitator | Which author/s conducted the interview or focus group? | 6 |
| 2. | Credentials | What were the researcher's credentials? *E.g. PhD, MD* | NR |
| 3. | Occupation | What was their occupation at the time of the study? | Title page |
| 4. | Gender | Was the researcher male or female? | Title page |
| 5. | Experience and training | What experience or training did the researcher have? | 17 |
| **Relationship with participants** | | | |
| 6. | Relationship established | Was a relationship established prior to study commencement? | 5 |
| 7. | Participant knowledge of the interviewer | What did the participants know about the researcher? e*.g. personal goals, reasons for doing the research* | Supplementary Material 1, Supplementary Material 2 |
| 8. | Interviewer characteristics | What characteristics were reported about the interviewer/facilitator? e.g. *Bias, assumptions, reasons and interests in the research topic* | 5, 7-8 |
| **Domain 2: study design** | | | |
| **Theoretical framework** | | | |
| 9. | Methodological orientation and Theory | What methodological orientation was stated to underpin the study? *e.g. grounded theory, discourse analysis, ethnography, phenomenology, content analysis* | 4 |
| **Participant selection** | | | |
| 10. | Sampling | How were participants selected? *e.g. purposive, convenience, consecutive, snowball* | 5 |
| 11. | Method of approach | How were participants approached? e*.g. face-to-face, telephone, mail, email* | 5 |
| 12. | Sample size | How many participants were in the study? | 8 |
| 13. | Non-participation | How many people refused to participate or dropped out? Reasons? |  |
| **Setting** | | | |
| 14. | Setting of data collection | Where was the data collected? e*.g. home, clinic, workplace* | 5-6 |
| 15. | Presence of non-participants | Was anyone else present besides the participants and researchers? | 5-6 |
| 16. | Description of sample | What are the important characteristics of the sample? *e.g. demographic data, date* | 8, Table 2, Table 3 |
| **Data collection** | | | |
| 17. | Interview guide | Were questions, prompts, guides provided by the authors? Was it pilot tested? | 5-6, Supplementary material |
| 18. | Repeat interviews | Were repeat interviews carried out? If yes, how many? | 6 |
| 19. | Audio/visual recording | Did the research use audio or visual recording to collect the data? | 5-6 |
| 20. | Field notes | Were field notes made during and/or after the interview or focus group? | 7 |
| 21. | Duration | What was the duration of the interviews or focus group? | 6 |
| 22. | Data saturation | Was data saturation discussed? | 6 |
| 23. | Transcripts returned | Were transcripts returned to participants for comment and/or correction? | 6 |
| **Domain 3: analysis and findings** | | | |
| **Data analysis** | | | |
| 24. | Number of data coders | How many data coders coded the data? | 6-7 |
| 25. | Description of the coding tree | Did authors provide a description of the coding tree? | 6-7 |
| 26. | Derivation of themes | Were themes identified in advance or derived from the data? | 6-7 |
| 27. | Software | What software, if applicable, was used to manage the data? | 6 |
| 28. | Participant checking | Did participants provide feedback on the findings? | 8 |
| **Reporting** | | | |
| 29. | Quotations presented | Were participant quotations presented to illustrate the themes / findings? Was each quotation identified? e*.g. participant number* | 9-14 |
| 30. | Data and findings consistent | Was there consistency between the data presented and the findings? | 9-14 |
| 31. | Clarity of major themes | Were major themes clearly presented in the findings? | 9-14 |
| 32. | Clarity of minor themes | Is there a description of diverse cases or discussion of minor themes? | 9-11 |
